# Bacterial Communities in Polluted Seabed Sediments: A Molecular Biology Assay in Leghorn Harbor

**DOI:** 10.1155/2013/165706

**Published:** 2013-10-08

**Authors:** Carolina Chiellini, Renato Iannelli, Franco Verni, Giulio Petroni

**Affiliations:** ^1^Department of Biology, Unit of Protistology-Zoology, University of Pisa, via Luca Ghini 13, 56126 Pisa, Italy; ^2^Department of Engineering for Energy, Systems, Territory and Constructions, University of Pisa, via Carlo Francesco Gabba 22, 56122 Pisa, Italy

## Abstract

Seabed sediments of commercial ports are often characterized by high pollution levels. Differences in number and distribution of bacteria in such areas can be related to distribution of pollutants in the port and to sediment conditions. In this study, the bacterial communities of five sites from Leghorn Harbor seabed were characterized, and the main bacterial groups were identified. T-RFLP was used for all samples; two 16S rRNA libraries and *in silico* digestion of clones were used to identify fingerprint profiles. Library data, phylogenetic analysis, and T-RFLP coupled with *in silico* digestion of the obtained sequences evidenced the dominance of *Proteobacteria* and the high percentage of *Bacteroidetes* in all sites. The approach highlighted similar bacterial communities between samples coming from the five sites, suggesting a modest differentiation among bacterial communities of different harbor seabed sediments and hence the capacity of bacterial communities to adapt to different levels and types of pollution.

## 1. Introduction

Sea stretches of commercial ports are often characterized by high levels of pollution in sediments, low oxygen concentrations in the water column, and low biodiversity of benthic communities [1] that may cause the decrease of fauna in their seabed [2]. Further reasons of impact on harbor sea stretches often come from high concentrations of organic matter due to eutrophication, time variability of sediment deposition, allochthonous inputs, and low hydrodynamism [3, 4]. These reasons demonstrate that the activities conducted in ports lead to critical conditions in the environments, and the organisms develop several ways to adapt to the alterations of water and sediments parameters [5]. A crucial characteristic of harbor sites is the removal of organic matter. Consumption capacity of organic matter is mainly due to biotic uptake and degradation performed by microorganisms. This process is generally insufficient to maintain the equilibrium of ecosystems; in most cases, the presence of detrital organic matter (which increases anoxic conditions), heavy metals, polychlorinated biphenyls (PCBs, [6]), and other toxic compounds plays also a crucial role in negatively impacting the seabed biocenosis [7, 8]. Although the origin and concentration of pollutants in seabed harbor sediments can be different, many studies demonstrate that, in most cases, pollution is mainly due to the presence of hydrocarbons, heavy metals [9], and organic matter. Possible methods for hindering the problem of contaminated aquatic sediments have been widely discussed [10]. Polluted seabed sediments can be sanitised via *in situ* actions, which always require an accurate knowledge of the local biocenosis [11]. Moreover, it was demonstrated that bacterial communities have great potential to be used as sensitive indicators of contamination in aquatic sediments [12]. New and accurate methods to study the composition of microbial communities in harbor seabed sediments are necessary for these reasons. Differences in number and distribution of bacteria, in such a large area as a harbor, can be related to pollutant distribution and general sediment state. Today, Leghorn's Harbor is one of the most important ports in the Mediterranean Sea, linked with more than 300 ports worldwide. It is a multipurpose port that can cater for all kinds of vessels and handle all kinds of goods, as well as passenger traffic. It sports a marine area of 1.6 km^2^ and a useable land area of 2.6 km^2^. The Harbor offers 11 km of quays with more than 90 berths, with up to 13 m draught. The total extension covers one km^2^ surface outdoor and 70000 m^2^ indoor areas.

In the present research, the bacterial community composition of Leghorn Harbor seabed sediments was studied with a molecular fingerprint approach and with the construction of libraries of the 16S rRNA gene. The Terminal Restriction Fragment Length Polymorphism (T-RFLP) approach was used for all samples to identify the community structure. *in silico *digestion of clones retrieved from two clone libraries (one for a less polluted sample and one for a more polluted sample) was used for the identification of bacterial Operational Taxonomic Units (OTUs) in the T-RFLP fingerprint profiles. The aim of the study was to highlight how the different conditions of port seabed influence the composition of microbial communities and provide hints of dynamics for nutrient removal.

## 2. Materials and Methods

### 2.1. Description of the Study Area: Collection and Storage of Samples

**Figure 1:**
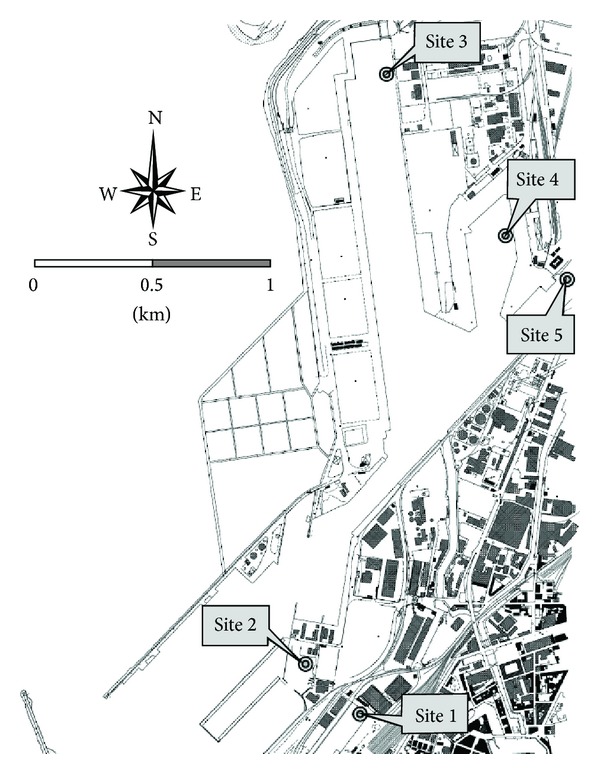
Representation of the Leghorn's Harbor area. The tagged marks represent the sampling sites in the seabed of the area, which are briefly described as follows. Site 1: ferry boat departure area (sandy loam; water depth 11 m). Site 2: seldom dredged shipbuilding area (loam; water depth 4 m; last dredging 60 years ago). Site 3: container terminal (silt loam; water depth 13 m; located by the open sea mouth of the Navicelli canal). Site 4: cargo ferry transit (loam; water depth 6 m). Site 5: chemical processing and oil refinery terminal (silty clay loam; water depth 9 m; located by the mouth of Ugione stream, which crosses the inland industrial area and collects some spare wastewater discharges).

Figure 1 shows Leghorn's Harbor area. The five sampling sites are located with tagged marks and briefly described within the figure. In each site, three samples were collected for a total of fifteen samples. According to the site assessment performed by the port authority [13] and the chemical parameters reported in Table 1, site one, which is located in the passenger terminal, represents the less polluted spot, while site two, which is located inside the “Darsena Calafatari” ship repairing section and has never been dredged for 60 years, has the highest concentrations of heavy metals and hydrocarbons. In this work, the samples named 2.1 (site 2, sample 1) and 1.2 (site 1, sample 2) were used. They were collected on the November 3, 2009, by a scuba diver and immediately brought to the laboratory for the extraction of the total DNA.

### 2.2. DNA Extraction, Construction of 16S rRNA Gene Libraries, Sequencing, T-RFLP Analysis, and *In Silico* Digestion of Sequenced Clones

Total DNA extraction was performed on all of the 15 samples using a soil master DNA extraction kit (Epicentre Biotechnologies, WI, USA). The library was built with samples 1.2 and 2.1 using the procedure described by Amann et al. [14]: 16S rRNA genes were directly amplified from extracted DNA using universal bacterial primers, 8F (5′-AGA GTT TGA T(CT)(AC) TGG CTC AG-3′) and reverse 1492R (5′-GG(AGCT)(AT)AC CTT GTT ACG ACT T-3′) [15]; the amplification products were cloned in a plasmid vector (pCRs2.1-TOPOs, TOPO TA Cloning Kit, Invitrogen, UK) and inserted in chemically competent cells (OneShot TOP10, Invitrogen, UK). The inserted fragments from a representative number of clones were then amplified by control PCR with M13F and M13R universal primers. The inserted genes showing a size similar to the 16S rRNA gene size were directly sequenced with primers M13F and M13R by the Macrogen Inc. sequencing service (Republic of Korea).

For T-RFLP analysis, the amplification of the 16S rRNA gene was performed with the same procedures of the library construction, and primer 8F was labeled with FAM fluorochrome (Applied Biosystem CA, USA). The template was digested with two different restriction endonucleases: BsuRI (GG^∧^CC, 0.2 u/*μ*L, Fermentas, Canada) and RsaI (GT^∧^AC⁡, 0.2 u/*μ*L, Fermentas). Digested DNA was precipitated with cold ethanol 100% to eliminate salts at 4°C and 10,000 RCF. For each reaction, a mix was prepared with 1.2 *μ*L of loading buffer (GeneScan 600 LIZ, Applied Biosystem), a maximum of 5.5 *μ*L of sample (calculated after cold ethanol precipitation on the bases of its final concentration), and 13.3 *μ*L of deionized formamide (Applichem, Germany). Capillary electrophoresis was performed with Abi Prism 310 Genetic Analyzer (Applied Biosystem); T-RFLP profiles were analyzed using GeneScan analysis software (Applied Biosystem), and the data matrix was transformed for statistics as described in Iannelli et al. [13]. Nonmetric Multidimensional Scaling (NMDS) was performed on the whole T-RFLP dataset with the Bray-Curtis coefficient and the Shannon diversity index was also calculated. All statistical analyses were performed using PAST software v.2.15 (Paleontological Statistic, [16]). Further details on the applied techniques are reported in Chiellini et al. [17]. All sequences obtained from the construction of both libraries were digested *in silico* by searching the restriction site on Arb database, as recognized by restriction enzymes BsuRI and RsaI in order to know the size of the terminal restriction fragments representative of the OTUs. With the *in silico* digestion of retrieved clones, we could correlate the peaks on T-RFLP fingerprints of the 15 samples from Leghorn Harbor seabed (3 replicates in 5 different sites) to specific OTUs. The coverage percentages of the peaks corresponding to the recognized OTUs and corresponding bacterial phyla and classes were calculated for each electropherogram.

### 2.3. Detection of Chimeric Sequences and Phylogenetic Analysis

All the retrieved sequences were scanned with the Bellerophon server [18] to identify chimeras. We decided to analyze chimeric sequences by “cutting” them in proximity of the recombination site identified by Bellerophon software and considering the obtained fragments as independent sequences belonging to different organisms. Fragments were treated in the same way as complete sequences from other screened clones; they were included in 16S rRNA library analysis, but they were not included in the construction of phylogenetic trees, due to their short length. NCBI BLAST analysis [19] was used to determine a preliminary affiliation of clone sequences. After BLAST analysis, sequences were inserted in SILVA 104 database [20] and aligned using the appropriate tool from the ARB program package [21]. A deeper phylogenetic analysis was performed on the *Proteobacteria* phylum, because (i) it included the most represented 15 T-RFLP profiles in the two libraries, and (ii) it was one of the main groups previously retrieved in marine sediments (e.g., [37, 38]). Two phylogenetic reconstructions on different subclasses of *Proteobacteria *were performed independently. The analysis focuses in particular on two groups of sequences: the first one including bacteria belonging to the *Deltaproteobacteria* subclass using *Epsilonproteobacteria* as an outgroup, and the second one focusing on the *Gammaproteobacteria* group with some sequences of *Alphaproteobacteria* used as an outgroup. Both trees were constructed using maximum likelihood algorithm, 100 bootstraps, and a filter specifically designed for the selection of sequences in each tree, considering only positions conserved in at least 10% of sequences. A distance matrix, constructed using the neighbor joining algorithm, was also calculated and examined for clone sequences of the two samples that clustered together with described and cultivated bacterial species, in order to understand which clades represented the species-level or the genus-level groups. In the construction and interpretation of similarity matrices, we used the 95% limit to represent the threshold for genus definition and the limit of 97% to represent the threshold for OTUs [22].

### 2.4. Statistical Analysis of Clone Libraries

OTUs were identified in each library using MOTHUR software [23]. Richness and alpha diversity indices, including the Chao 1 estimator [24] and the Shannon index, were calculated for each library at different cutoff levels (0.01, 0.03, and 0.05).

The Chao 1 estimator evaluates the richness of a total species as

(1) $$\begin{array}{c} \text{Chao}\;\;1=\text{Sobs}+\frac{n_{1}^{2}}{2n_{2}}, \end{array}$$

where Sobs is the number of observed species, *n*_1_ is the number of singletons (species captured once), and *n*_2_ is the number of doubletons (species captured twice).

Shannon's diversity index [25] was obtained as

(2) $$\begin{array}{c} H=-\Sigma p_{i}\ln p_{i}, \end{array}$$

where *p*_*i*_ is the population of each species *i*, and the resulting product is summed up across species and multiplied by −1 [26].

The MOTHUR software was also applied to calculate the number of OTUs shared among the two different libraries at different cutoff levels. The LIBSHUFF software [27], based on the Jukes-Cantor pairwise distance matrix, was also applied to find out similarities in the two libraries, as described by Zhang et al. [28]. 

## 3. Results

### 3.1. Construction of 16S rRNA Gene Libraries and Detection of Chimeric Sequences

**Figure 2:**
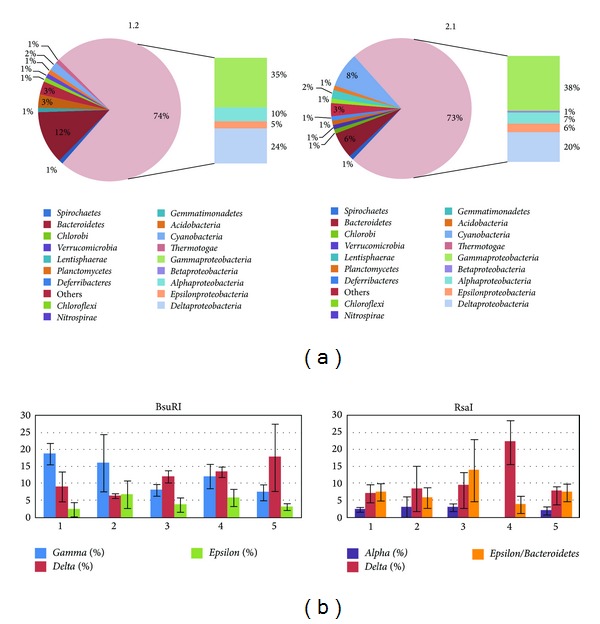
(a) Comparison among main bacteria phyla detected in the two clone libraries; the larger pink sectors represent *Proteobacteria.* (b) Coverage percentages of the main bacterial groups attributed through *in silico* digestion of the 15 T-RFLP electropherograms. Each of the five sites is represented by the means of three replicates; the black bars represent the standard deviations.

Two libraries were constructed on samples 1.2 and 2.1. A total of 194 clones were sequenced, 101 of which taken from sample 1.2 and 93 from sample 2.1. All obtained sequences were submitted online on the DDBJ/EMBL/GenBank databases. They are available under the accession numbers from HE803828 to HE804037. The percentages of detected chimeric sequences were 11.9% in sample 1.2 (12 sequences) and 4.3% in sample 2.1 (4 sequences). All chimeric sequences were composed of partial sequences coming from only two different organisms. Considering fragments constituting chimeras as independent sequences belonging to different organisms, the library from sample 1.2 was composed of a total of 115 sequences and the library from sample 2.1 of 95 sequences. The affiliation of nucleotide sequences was determined by BLAST analysis. In both libraries, the highest percentage of screened clones emerged as belonging to the *Proteobacteria* phylum (74% to library 1.2 and 73% to library 2.1, Figure 2(a)). Within this group, bacteria belonging to *Alpha-*, *Gamma-, Delta-,* and *Epsilonproteobacteria* classes were present in all libraries, while bacteria belonging to the *Betaproteobacteria* class were detected only in library 2.1 with low percent values (1%). In particular, the *Gammaproteobacteria* subclass was the most represented one in both 1.2 and 2.1 libraries (35% and 38%, resp.), followed by the *Deltaproteobacteria* subclass (resp., 24% and 20%). The second most represented bacterial taxon detected in both libraries was the *Bacteroidetes* phylum, represented by 12% of the total sequences in library 1.2, and by 6% of the sequences in library 2.1. There are some differences in the distribution of bacterial phyla detected in the two libraries, especially for minor groups such as the *Lentisphaerae*, *Nitrospirae*, and *Thermotogae* phyla, that were present only in sample 1.2 with percent values lower than 1%, and the *Chlorobi*, *Verrucomicrobia*, *Deferribacteres*, and *Gemmatimonadetes* phyla, that were detected in sample 2.1 with fractions ranging from 1% to 2%.

### 3.2. T-RFLP Analysis

**Figure 3:**
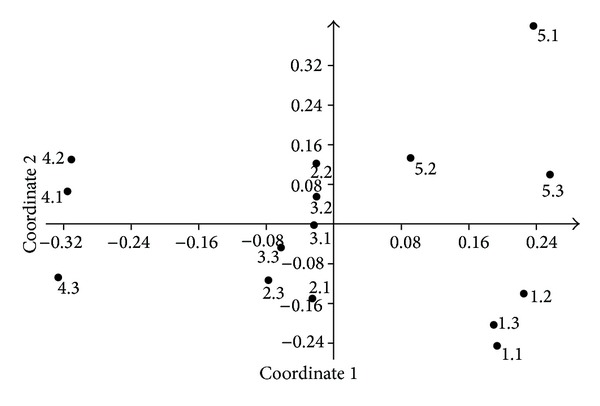
NMDS plot obtained from T-RFLP data analysis.

NMDS analysis on T-RFLP data matrix highlights some groups of samples that correspond to the five sampling sites (Figure 3). The grouping of the triplicates is mainly evident for sites 1 (samples 1.1, 1.2, and 1.3) and 4 (samples 4.1, 4.2, and 4.3). Triplicates of sites 2 and 3 clustered together in the central part of the plot, and the triplicates from site 5 (samples 5.1, 5.2, and 5.3) were all located in the second quadrant of the plot, isolated from all the other samples.

**Figure 4:**
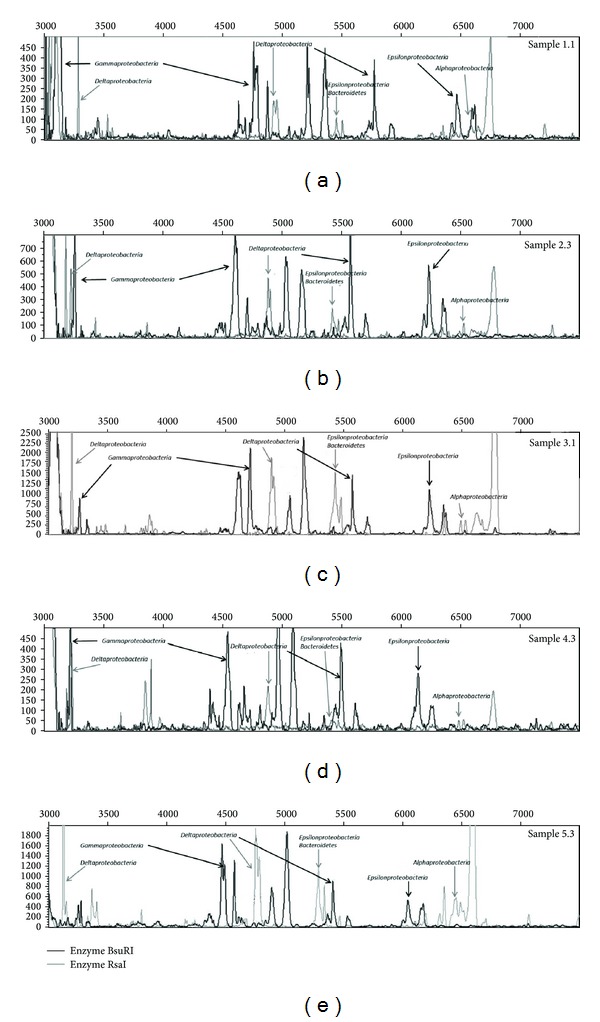
T-RFLP peak attribution after *in silico* digestion.

The Shannon diversity index calculated from the T-RFLP data matrix ranged from 3.17 to 3.75.  Figure 4 presents some of the results of the attribution of the peaks obtained by *in silico* clone digestion, in comparison with the peaks detected in the microbial communities of the five sampling sites. The coverage percentages of the bacterial groups in T-RFLP profiles are shown in Figure 2(b). Not all the bacterial OTUs detected with the rDNA clone library could be retrieved in the T-RFLP electropherograms of the 15 samples. All OTUs retrieved in the T-RFLP electropherograms through *in silico* digestion belong to subclasses of the* Proteobacteria* phylum, with the only exception of the *Betaproteobacteria* class, which has not been detected. Figure 2(b) highlights a dominance of *Gammaproteobacteria* in sites 1 and 2 and a dominance of *Deltaproteobacteria* organisms in sites 3, 4, and 5. The less represented group of *Proteobacteria* was the *Epsilonproteobacteria* in all BsuRI samples. When considering the RsaI restriction endonuclease, the *Alphaproteobacteria* subclass emerged as the less represented group in each case.

### 3.3. Phylogenetic Analysis

**Figure 5:**
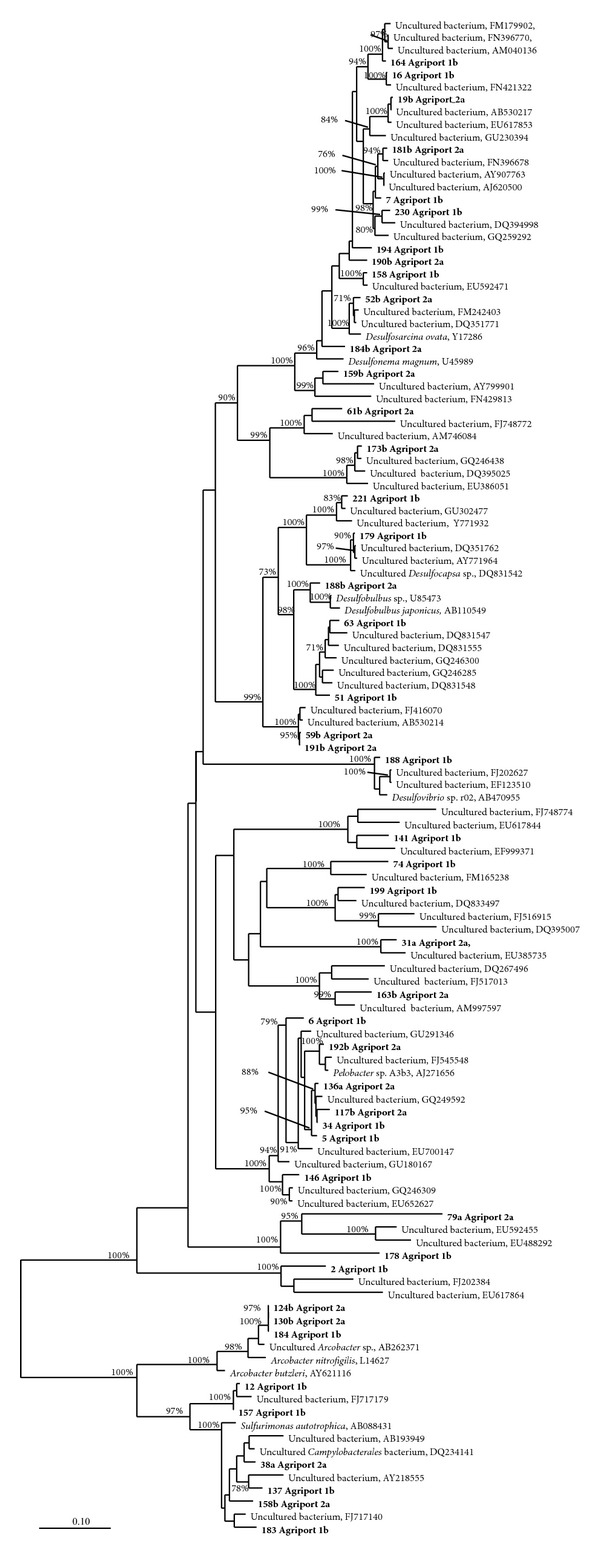
Maximum likelihood phylogenetic tree made with PHYML and 100 bootstrap pseudoreplicates. The tree represents the phylogenetic position of characterized *Delta-* and *Epsilonproteobacteria* clone sequences together with closely related sequences present in the database. The characterized sequences are in bold.

**Figure 6:**
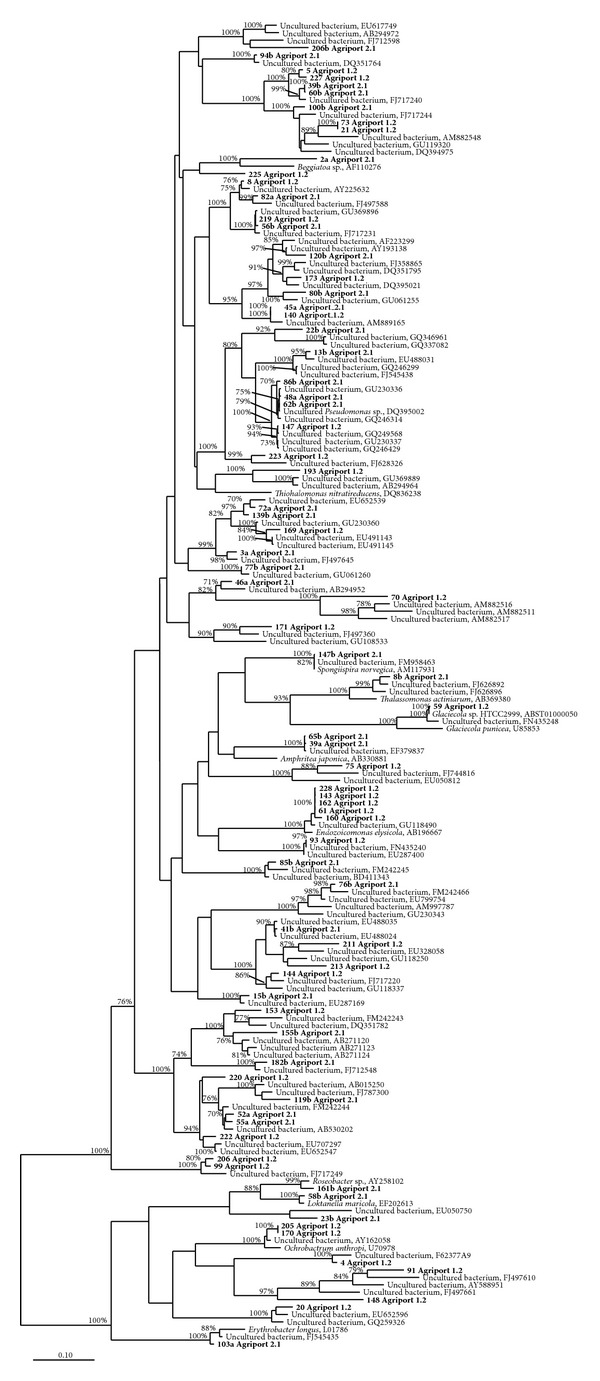
Maximum likelihood phylogenetic tree made with PHYML and 100 bootstrap pseudoreplicates. The tree represents the phylogenetic position of characterized *Alpha-* and *Gammaproteobacteria* clone sequences together with closely related sequences present in the database. The characterized sequences are in bold.

In order to assess the relative abundance of *Proteobacteria *related sequences, we focused on the phylogenetic analysis of this group of organisms. In particular, two phylogenetic trees were built: the first one comprised all the retrieved *Delta-* and *Epsilonproteobacteria* clone sequences and their closer relatives (Figure 5); the second one included all the retrieved *Gamma-* and *Alphaproteobacteria* clone sequences and their closer relatives (Figure 6). The trees were constructed using PHYML with 100 bootstraps [29] from the ARB package. The majority of sequences in the trees are derived from studies about marine sediment samples. Some of our clones are closely related to symbiotic bacteria found in marine metazoan bodies [30, 31]. These sequences belong to the *Gammaproteobacteria* subclass; clone 147b from library 2.1 shows a 99% similarity with the already mentioned *Spongiispira norvegica* (AM117931, [31]), while the OTU composed of clones 228, 143, 162, 61, and 160, all coming from library 1.2, shows a 98% similarity with *Endozoicomonas elysicola* (AB196667, [30]). All the clones related to the *Deltaproteobacteria* subclass cluster together with species having a metabolism involving the presence of sulphur (most sulphate reducing microorganisms). The majority of published sequences that are closely related to our clone sequences belong to uncultured bacteria, with the exception of some *Deltaproteobacteria*, which belong to *Desulfobulbus*, *Desulfosarcina*, and *Desulfonema* genera, all providing the sulfate reduction in the marine environment. The similarity matrix evidenced that within the *Deltaproteobacteria* subclass our clone 188b from library 2.1 showed a 95% similarity with the described *Desulfobulbus japonicus* species (AB110549, [32]), while the 52b clone from library 2.1 showed a 96% similarity with the described *Desulfosarcina ovata *(Y17286, [33]). The 124b and 130b sequences from library 2.1 and the sequence 184 from library 1.2 are all parts of the same OTU. They cluster together with the described *Epsilonproteobacteria* species *Arcobacter nitrofigilis* (L14627, [34]), symbiotic bacteria characterized by a nitrogen fixing metabolism.

### 3.4. Statistical Analysis of Clone Libraries

Table 2 shows the results concerning richness and alpha diversity indices in each of the two constructed libraries at three different cutoff levels. The Shannon diversity index shows similar values in both sampling sites, at all cutoff levels. The richness index (Chao1 estimator) emerged as being higher in sample 1.2 at 0.01 cutoff level and lower in sample 1.2 at 0.03 and 0.05 cutoff values. The number of detected OTUs emerged as being very similar in both sites. The OTUs at a 0.03 cutoff value were represented by the same number in both cases; library 2.1 at 0.01 and 0.05 cutoff values shows less detected OTUs than library 1.2 at the same cutoff levels (Table 2).

Table 3 presents the MOTHUR analysis of OTUs that are shared between the two different libraries. At 0.01 cutoff (specie level), there are 7 OTUs shared between sites 1.2 and 2.1; at the 0.03 cutoff there are 12 shared OTUs; at 0.05 cutoff, the number of shared OTUs, which usually represents the genus level, rises up to 17.

**Figure 7:**
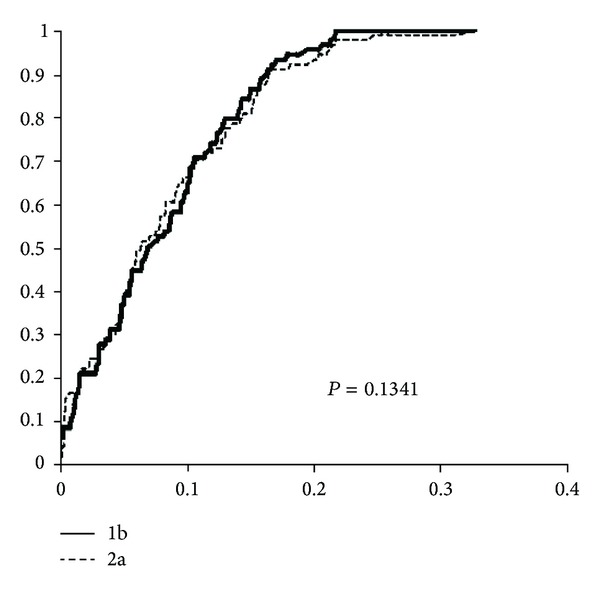
Results of LIBSHUFF comparison between the 1.2 and 2.1 libraries. The solid line indicates the homologous coverage curve *C*_*X*_(*D*), and the broken line indicates the heterologous coverage curve *C*_*XY*_(*D*). The *P* value is *P* > 0.025, thus indicating that the two libraries are similar to each other in OTU composition. The *y*-axis represents the coverage (*C*) while the *x*-axis represents the evolutionary distance (*D*).

Figure 7 presents the results of the LIBSHUFF analysis. The two lines, representing the homologous and heterologous curves, are almost totally overlapping, thus indicating that the samples are similar to each other, as confirmed by the high *P* value (*P* > 0.025 with Bonferroni correction, as described in [27]).

## 4. Discussion

The molecular approach is commonly used in the recent literature for the study of bacterial communities in marine sediments [35–38] and to study the influence of bacterial communities in removing pollutants from marine sediments [39]. The two libraries highlighted a substantial similarity concerning the abundance and diversity of the sequences represented in the two different sites, even though the chemical composition was different [13]. The similarity between the two clone libraries is shown by the alpha diversity indices calculated with MOTHUR software (Table 2). The Shannon diversity index calculated for both libraries at different cutoff levels, on the base of the DNA sequences obtained after the library construction, ranged from 4.07 to 4.36. The same Shannon diversity index calculated on the base of T-RFLP profiles of the 15 samples ranged from 3.17 to 3.75. This difference can be explained because the diversity in a sample is underestimated when only T-RFLP profiles are used. This approach does not register rare OTUs. The Shannon diversity index used in this work should provide more reliable values, although an increase of the sample size of the sequenced clones of each library could probably provide even more accurate values.

The construction of the two libraries and the screening of clones coupled with T-RFLP peak identification highlighted the dominance of *Proteobacteria* related microorganisms in all the five sites. There are no significant differences among the coverage percentages of different subclasses of the *Proteobacteria* phylum among the two libraries and the 5 sites; these data are in agreement with previously published studies, where PCR-based techniques on marine sediment samples revealed a dominance of bacteria related to *Proteobacteria* phylum [35, 37] or to *Firmicutes*, *Delta-*, and *Gammaproteobacteria *[40, 41]. In other papers, the analysis of marine sediment showed a dominance of *Actinobacteria* [38]. In this study, no significant differences in the coverage percentage of different subclasses of the *Proteobacteria* phylum were observed among the five sites; this agrees with other studies in which different libraries of marine sediments collected in different sites showed similar bacterial compositions [35]. The *Bacteroidetes* phylum is the only further phylum with an appreciable presence in all sites, and the percentages of abundance are comparable to those found by other authors [35, 38]. This result can mean that bacteria belonging to these groups play the main role in nutrient recycling in the harbor seabed ecosystem.

Considering the digestion profiles obtained with the BsuRI restriction endonuclease, *Gammaproteobacteria* emerged as being dominant in sites 1 and 2, while the sediments were dominated by *Deltaproteobacteria* organisms in sites 3, 4, and 5. Considering the RsaI restriction endonuclease, sites 2, 4, and 5 emerged as being dominated by *Deltaproteobacteria* microorganisms, while sites 1 and 3 were dominated by *Epsilonproteobacteria* and *Bacteroidetes*, which are groups of bacteria sharing the same peak interval. Some groups of bacteria could be detected only with one of the two adopted restriction enzymes. For instance, *Alphaproteobacteria* were not detected with BsuRI and *Gammaproteobacteria* were not detected with RsaI. This circumstance can be explained by the fact that the first restriction site in the gene sequences of those bacteria is located outside the interval 50–500 bp, which is the interval covered by our T-RFLP analysis. In fact, the use of more than one restriction enzyme is a recommended practice in this technique, in order to facilitate the resolution of bacterial populations [42, 43]. The fact that other OTUs identified through clone library construction were not identified in T-RFLP profiles can be in part explained by their poor representation in the sediment samples, causing the failure of their T-RFLP quantification.

Overall, our results are in agreement with results of other authors that examined samples of port sediments. In the study performed by Wu et al. [44], three different samples of marine sediments were investigated in order to characterize their bacterial communities. In all samples, the dominant phylum was *Proteobacteria* with a presence of 82%, 42%, and 42% in the three samples. Zhang et al. [28] analyzed four sites characterized by different pollution levels in Victoria Harbor (Hong Kong). In all sites, they found the dominance of bacteria belonging to the *Proteobacteria* phylum and, especially, to the same subclasses detected in our work. The dominance of *Proteobacteria* was also found in many other studies (e.g., [37, 45, 46]). The diversity index calculated by Zhang et al. [28] did not highlight significant differences among the four analyzed sites; this result is similar to the finding of this work, where the two analyzed libraries highlighted similar Shannon diversity values. Bacteria belonging to the *Alphaproteobacteria* subclass are important for hydrocarbon degradation [47]. The presence of these bacterial taxa in all five sites confirms a contamination of hydrocarbons in Leghorn seabed sediments [13].

A large portion of the sequences detected in our clone libraries belong to the *Deltaproteobacteria* subclass. With a deep phylogenetic analysis, we discovered that all these clones are closely related to bacteria characterized by a metabolism involved in the removal of sulphur. This observation is in agreement with other two works performed on marine port sediments [28, 48]. The percentage of *Deltaproteobacteria* detected in the two different samples is not significantly different (24% library 1.2 and 20% library 2.1), and T-RFLP results suggest that their abundance should be roughly comparable among all the five sites. This result does not agree with the chemical results published in Iannelli et al. [13], which evidenced that the concentration of SO_4_^2−^ in site 2.1 (4399 mg/(kg dw)) was double that in site 1.2 (2321 mg/(kg dw)).

An interesting finding concerns the *Gammaproteobacteria* subclass, in particular the sequences 228, 143, 162, 61, and 160, from library 1.2, and the sequence 147b from library 2.1. The first group of sequences, all representing the same OTU, shows a 98% similarity with *Endozoicomonas elysicola*, which is a typical symbiont of the *Elysia ornata* slug. Conversely, the sequence 147b is 99% similar to the sequence of *Spongiispira norvegica*, a typical symbiont of sponges. This observation possibly suggests that site 1.2 is richer in marine metazoans characterized by a symbiotic association with bacteria than site 2.1. This difference is probably due to the chemical characteristics of the sediments in Leghorn seabed area. Site 1.2 in fact, emerged as being less polluted than site 2.1, and probably more suitable for being colonized by marine metazoans harboring bacterial symbionts. 

## 5. Conclusions

In the present study, bacterial communities from five sites of Leghorn Harbor seabed were analyzed and identified through T-RFLP analysis, 16S rRNA library construction, and *in silico* digestion of retrieved clones. Some considerations emerged about organisms involved in the recycling of organic matter. 

Both the diversity indices and the phylogenetic affiliation of bacterial sequences obtained from the molecular screening highlighted a substantial similarity in the composition of the bacterial communities. This finding contrasts with the chemical characterization of an earlier study, where the same sites evidenced significant differences in the presence of nutrients and pollutants, and the T-RFLP analysis evidenced a heterogeneity among the different sites of the harbor.

Retrieved sequences from the two libraries were more than sufficient to provide generic information on metabolism present in all of the 15 seabed sediment samples and to compare them with previous similar studies. Only a significantly higher sequencing coverage would have probably allowed to distinguish between the samples that looked rather similar in the presented analysis. The T-RFLP approach proved to be more efficient in highlighting the bacterial community structure in the whole harbor area, whereas the present work helped in clarifying the role of specific bacteria present in the studied samples and their related metabolisms. 

## Figures and Tables

**Table 1:** Chemical parameters of the five sampling sites [13].

Samples	Cd	Ni	Pb	Cr	Cu	Zn	TPH	TOC	TN	TP	NO_3_^−^	Cl^−^	SO_4_^2−^
	mg/kg_dw_	mg/kg_dw_	mg/kg_dw_	mg/kg_dw_	mg/kg_dw_	mg/kg_dw_	mg/kg_dw_	mg/kg_dw_	mg/kg_dw_	mg/kg_dw_	mg/kg_fw_	g/kg_fw_	mg/kg_fw_
1.1	1.06	37.7	33.2	25.2	75.0	313	1433	6102	197	917	831	10	3587
1.2	0.63	25.2	271.0	34.7	73.5	353	1066	8938	225	1024	975	12	4211
1.3	0.71	39.6	104.0	15.8	268.0	407	1030	7720	304	1321	903	11	3899

2.1	1.16	41.0	249.0	48.8	80.5	308	1915	25460	2170	696	1161	17	3192
2.2	1.42	75.4	221.0	35.4	375.0	854	2031	33540	1580	759	1363	19	3747
2.3	1.31	94.9	171.0	27.7	485.0	884	1798	27500	1890	722	1262	18	3469

3.1	1.22	101.0	47.1	32.1	74.4	257	900	19664	1019	568	708	16	3417
3.2	1.44	98.4	36.5	35.6	71.5	336	766	25736	1325	651	832	19	4012
3.3	1.20	118.0	37.8	29.2	71.4	228	633	21200	1631	455	770	17	3714

4.1	1.09	63.1	58.4	16.9	123.0	568	1765	19572	730	616	1929	17	3848
4.2	1.20	67.0	54.3	18.7	91.3	277	1563	21100	878	504	2265	20	4517
4.3	1.11	67.4	90.0	25.0	86.4	247	1666	16628	602	570	2097	18	4183

5.1	1.36	100.0	62.0	34.6	119.0	500	1266	17148	1993	864	491	17	2807
5.2	1.59	103.0	65.6	39.4	127.0	476	1331	26652	1660	724	576	20	3295
5.3	1.48	135.0	67.7	42.6	135.0	368	1032	23900	1327	844	534	19	3051

TPH: total petroleum hydrocarbons; TOC: total organic carbon; TN: total N; TP: total P; dw: dry weight; fw: fresh sample weight. All parameters are means of three replicates.

**Table 2:** Alpha diversity indices for the two samples at different cutoff values.

Sample	cutoff	Observed richness (OTUs)	Chao 1 estimator	Shannon index
1.2	0.01	82	667,2	4,36
	0.03	75	258,3	4,24
	0.05	71	193,8	4,17

2.1	0.01	80	620,2	4,33
	0.03	75	306,1	4,24
	0.05	68	239,1	4,07

**Table 3:** OTUs shared between site 1.2 and 2.1 calculated with MOTHUR software.

Cutoff	Observed richness shared between 1.2 and 2.1
0.01	7
0.03	12
0.05	17
